# Evaluation of acute, repeated dose 28-day and 13-week oral toxicity and genotoxicity of a standardized fraction (HemoHIM) from *Angelica gigas, Cnidium officinale, and Paeonia lactiflora*

**DOI:** 10.1007/s43188-024-00252-1

**Published:** 2024-08-16

**Authors:** Ju Gyeong Kim, Su-Bin Bak, Gyoung-Deuck Kim, Han-Sol Choi, Da-Ae Kwon, Ha-Young Kim, Dong-Won Son, Jang-Hun Jeong, Byung-Woo Lee, Hyo-Jin An, Hak Sung Lee

**Affiliations:** 1 Food Science R&D center, Kolmar BNH Co., Ltd., 61, Heolleung-Ro 8-Gil, Seocho-Gu, Seoul, Republic of Korea; 2 Biotoxtech CO. Ltd., 53, Yeongudanji-Ro, Ochang-Eup, Cheongwon-Gu, Cheongju-Si, Chungcheongbuk-Do 28115 Republic of Korea

**Keywords:** HemoHIM, Functional food, Acute toxicity, Repeated dose toxicity, Genotoxicity

## Abstract

HemoHIM is a functional food ingredient comprising a triple herbal combination of extracts from *Angelica gigas* Nakai, *Cnidium officinale* Makino, and *Paeonia lactiflora* Pallas. It was developed to aid the recovery of impaired immune function. Although it is widely used to treat various immune disorders in Korea, its potential toxicity has not been extensively investigated. Therefore, a comprehensive study was conducted to assess the safety of HemoHIM, including acute oral dose toxicity, 28-day and 13-week repeated-dose toxicity, and genotoxicity. To evaluate its safety profile, the dose was increased to 2,000 mg/kg/day, which corresponds to the dose limit for acute toxicity as per the Organization for Economic Cooperation and Development Test Guideline 423. No abnormal findings were observed at the higher doses. For the 28-day and 13-week repeated-dose toxicity studies, HemoHIM was administered at doses of 500, 1,000, and 2,000 mg/kg/day to examine subchronic toxicity in male and female rats. No test item-related clinical signs or mortality was observed at any of the tested doses. Gross pathology, hematology, blood chemistry, and histopathology evaluations further supported the safety of HemoHIM. Therefore, the NOAEL of HemoHIM was considered to be at 2,000 mg/kg/day for both sexes of rats. Bacterial reverse mutation tests, a chromosome aberration test in human peripheral blood lymphocytes, and a mouse micronucleus test were conducted to determine the genotoxicity of HemoHIM, which revealed that HemoHIM was non-mutagenic and non-clastogenic. Collectively, these findings provide valuable evidence to support the safe use of HemoHIM as a functional food ingredient.

## Introduction

In traditional Oriental medicine, numerous herbs and herbal prescriptions have long been recognized for their potential to promote health, fortify the body’s defense mechanisms, and contribute to longevity [1]. HemoHIM (MFDS Recognition Number: 2006–17) is derived from an extract containing a polysaccharide fraction obtained from three distinct herbs: *Angelica gigas* Nakai, *Cnidium officinale* Makino, and *Paeonia lactiflora* Pallas [2, 3]. These herbs are acknowledged as raw materials in the Korea Food Code. HemoHIM is formulated by blending their polysaccharide fractions in a standardized specific ratio. The chemical constituents of HemoHIM include chlorogenic acid, paeoniflorin, and nodakenin [4, 5].

Chlorogenic acid, a non-flavonoid polyphenol abundant in plant leaves, possesses antiviral, antibacterial, antimutagenic, antiphlogistic, and antioxidant properties, protecting against chemical-induced toxicities [6]. Paeoniflorin, a monoterpene glucoside and the primary active component of Paeonia Radix [7], exhibits antioxidant, anti-inflammatory, and neuroprotective effects in various cells [8, 9]. It also induces the expression of heat shock proteins and protects against stress [10]. Nodakenin, derived from *Angelica gigas* roots, treats various disorders, inhibits allergic inflammation by downregulating NF-κB and Caspase-1 activation [11, 12], and relieves renal ischemia–reperfusion injury by inhibiting reactive oxygen species-stimulated NLRP3 inflammasome activation [13]. These bioactive compounds have significant therapeutic potential, highlighting their importance in medicinal applications.

Considering the impressive effects of these herbal ingredients, HemoHIM is a potential treatment and preventive measure against numerous diseases. It has also been shown to exert antitumor effects [14] and protect against H_2_O_2_-induced oxidative stress [15], and radiation-induced damage to the gastrointestinal and immune hematopoietic systems [16]. Furthermore, HemoHIM has exhibited various immune-enhancing effects, such as immune cell activation, immune hematopoietic recovery, tissue regeneration, and antioxidant activity [5, 16, 17].

Despite studies exploring the diverse physiological activities of HemoHIM, only a limited number have investigated its in vivo toxicity. Understanding the potential risks to human health associated with HemoHIM is crucial and requires further investigation. Consequently, this study aimed to evaluate the acute and subacute toxicity of orally administered HemoHIM in rats, and its in vitro and in vivo genotoxicity.

## Materials and methods

### Test items

HemoHIM, an extract of a triple herbal combination of *Angelica gigas* Nakai, *Cnidium officinale* Makino, and *Paeonia lactiflora* Pallas, contains chlorogenic acid (25–60 mg/100 g), paeoniflorin (200–400 mg/100 g), and nodakenin (50–150 mg/100 g). The sources are purchased from GEUNONONGLIM Agricultural Co. (Yeoju, Korea). It was manufactured by Kolmar BNH Co. Ltd. (Sejong, Korea). HemoHIM was prepared following the method detailed in our previous report [5]. Briefly, *Angelica gigas, Cnidium officinale, and Paeonia lactiflora* were extracted in equal amounts for 4 h in boiling water to obtain the extract. Half of this extract was then precipitated with ethanol to yield a water-soluble polysaccharide fraction. Subsequently, the HemoHIM was obtained by adding this polysaccharide fraction to the remaining half of the extract, which was concentrated to a solid content of 30% ± 3%. The product was then lyophilized using a freeze-dryer (FDU-1110, EYELA, Japan) and stored in a refrigerator at 4 ℃. No additional additives were introduced during this process. The formulation was prepared immediately before administration, specifically on the day of administration.

### Animals and husbandry

All rats (N Tac: SD, 7 weeks old) for acute and 28-day repeated dose oral toxicity studies were obtained from Vivo Bio tech Ltd. (Hyderabad, Telangana, India). Environmental conditions in the animal room were maintained as follows: temperature = 19.6–23.6 °C, relative humidity = 50–62%, air exchange rate 14 changes/h, and light/dark cycle = 12 h/12 h. Variations in these conditions had no effect on the study outcomes. All rats (Crl:CD(SD), 6 weeks old) for 13-week repeated dose oral toxicity study were obtained from Orientbio Inc. (Seongnam, Korea). Environmental conditions in the animal room were maintained as follows: temperature = 19–25 °C, relative humidity = 30–70%, air exchange rate 10–15 changes/h, and light/dark cycle = 12 h/12 h. Variations in these conditions had no effect on the study outcomes. All mice (Swiss albino, 8–10 weeks old) for in vivo micronucleus test were obtained from Mahaveera Enterprises (Hyderabad, Telangana, India). Environmental conditions in the animal room were maintained as follows: temperature = 19.6–22.8 °C, relative humidity = 52–61%, air exchange rate 14 changes/h, and light/dark cycle = 12 h/12 h. Variations in these conditions had no effect on the study outcomes.

### Acute oral toxicity

An acute oral toxicity study was conducted in accordance with the Organization for Economic Cooperation and Development (OECD) Guideline 423 [18], and OECD principles of Good Laboratory Practice (GLP) [C (97) 114/Final]. The acute toxicity of HemoHIM was assessed in male and female Sprague–Dawley rats via oral gavage, with the test item suspended in water, and administered up to the OECD dose limit (2,000 mg/kg of body weight). All animals were fasted overnight (14 h) (water ad libitum with no feed) prior to the administration of the test item. All animals were observed for mortality, morbidity, and signs of toxicity (clinical signs) at 30 min, and 1, 2, 3, and 4 h after dosing on day 0 and once daily thereafter for 14 days. Body weights were recorded prior to dosing on days 0, 7, and 14. At the end of 14 days observation period, necropsy and gross pathological examinations were performed.

### 28-Day repeated dose oral toxicity

A repeated-dose oral toxicity study was conducted in accordance with the OECD Guideline 407 [19], and OECD principles of GLP [C (97) 114/Final]. The doses were administered orally to Sprague–Dawley rats for 28 consecutive days, followed by a 14-day recovery period to assess the reversibility of any toxic effects. The test item was weighed, suspended in water, and administered to rats through the oral (gavage) route using a disposable syringe with a rat intubation cannula at dose levels of 500 mg/kg/day for low-dose (G2), 1,000 mg/kg/day for mid-dose (G3), 2,000 mg/kg/day for high-dose (G4), and high-dose recovery (G4R) groups. Rats in the control (G1) and control recovery (G1R) groups received only water. The administered dose volume was 10 mL/kg/day. Each group comprised five rats of each sex. Vehicle or test formulations were administered to each rat group once daily for 28 consecutive days. After 28 days of treatment, the administration of vehicle and test item dose preparation to the control recovery (G1R) and high-dose recovery (G4R) groups was discontinued, and the potential reversibility or persistence of any toxic effects was observed for 14 days. The animals were observed twice daily for mortality/morbidity and once daily for cage-side clinical signs. Detailed clinical examinations were performed once prior to the initiation of treatment and thereafter at weekly intervals and at the end of the treatment and recovery periods. The rats were observed once per week for changes in body weight and feed consumption. Hematological and clinical chemistry investigations, and measurement of organ weight were performed at the end of the treatment and recovery periods. Animals were subjected to detailed necropsy at the end of the treatment and the recovery periods and the organs specified in the study plan were collected and weighed. The relative organ weights were calculated as percentage of body weight. Histopathological examination was carried out on all collected organs from control (G1) and high dose (G4) groups.

### 13-Week repeated dose oral toxicity

A repeated dose 13-week oral toxicity study was conducted in reference to OECD Guideline 408 [20], and in accordance with OECD principles of GLP (as revised 1997) ENV/MC/CHEM(98)17. The doses were administered orally to Sprague–Dawley rats for 13 consecutive weeks. The test item was weighed, suspended in water, and administered to rats through the oral (gavage) route using a disposable syringe with a rat intubation cannula at graduated dose levels of 500 mg/kg/day for low-dose (G2), 1,000 mg/kg/day for mid-dose (G3), and 2,000 mg/kg/day for high-dose (G4). The rats in the control (G1) received only water. The administered dose volume was 10 mL/kg/day. Each group comprised 10 rats of each sex. Vehicle or test formulations were administered to each rat group once daily for 13 consecutive weeks. During the observation period, clinical and detailed clinical signs, measurement of body weight and food consumption, ophthalmological examinations, and urinalysis were observed. At the end of the observation period, hematological and clinical chemistry examinations, observation of the estrus cycle, organ weight measurements, gross postmortem examinations, and histopathological examinations were performed. Histopathological examination was performed as follows: Organs and tissues from all animals of the control group (G1) and high dose group (G4); and organs and tissues from dead animal in the low dose group. Histopathological examination was performed on the following organs: brain; Pituitary gland; thyroid gland; parathyroid gland; thymus; lung with bronchus; heart; aorta; trachea; spleen; liver; adrenal gland; kidney; salivary gland (submandibular, sublingual, parotid); esophagus; stomach; duodenum; jejunum; ileum with Peyer’s patch; cecum; colon; rectum; pancreas; testis; epididymis; prostate gland; seminal vesicle; coagulating gland; ovary; uterus with cervix; vagina; urinary bladder; sciatic nerve; mesenteric lymph node; submandibular lymph node; eye; optic nerve; Harderian gland; skin (inguinal); mammary gland (inguinal); bone (sternum, femur); bone marrow (sternum, femur), skeletal muscle (biceps femoris); spinal cord (cervical, lumbar, thoracic).

### Bacterial reverse mutation assay

An in vitro bacterial reverse mutation assay was conducted in accordance with the OECD Guideline 471 [21]. In the preliminary cytotoxicity assay, TA100 of *Salmonella typhimurium* was treated with the test item at 156.3, 312.5, 625.0, 1250.0, 2500.0, and 5000.0 μg/plate with (5% v/v, S9) or without metabolic activation. Vehicle and positive controls were maintained concurrently with the treatment groups. Based on the results observed in the preliminary cytotoxicity assay, 5000.0 μg/plate was selected as the highest concentration for the mutagenicity assay. Mutagenicity assays were performed using the TA1537, TA1535, TA98, and TA100 strains of *S. typhimurium* and WP2*uvr*A of *Escherichia coli*. The bacterial strains were treated with the test item at 312.5, 625.0, 1250.0, 2500.0, and 5000.0 μg/plate with (5% v/v, S9) or without metabolic activation. HemoHIM showed no genotoxic activity in this assay.

### In vitro mammalian chromosomal aberration assay

An in vitro mammalian chromosomal aberration assay was conducted in accordance with the OECD Guideline 473 [22]. Based on the results of the preliminary cytotoxicity assay, a chromosome aberration assay was conducted using test item concentrations of 156.25, 312.5, and 625 μg/mL with or without metabolic activation. Cyclophosphamide (with metabolic activation S9) and ametycin (without metabolic activation S9) were employed as clastogenic positive controls. Human blood lymphocytes were cultured using RPMI 1640 medium supplemented with 10% fetal bovine serum (FBS), 1% penicillin–streptomycin, and 2% phytohemagglutinin in a CO_2_ incubator at 37 ± 1 ℃ and 5 ± 0.5% CO_2_. These cultures were exposed to different test item concentrations for the short-term (4 h) and continuous exposure (22 h) groups. In short term exposure, after 4 h of treatment, the culture media with the test item was replaced with RPMI 1640 complete medium and incubated for 18 h at 37 ± 1 ℃ and 5 ± 0.5% CO_2_. For continuous exposure, cultured cells were treated with different test item concentrations for 22 h. After 22 h, cultures from the short-term and continuous exposure groups were harvested and processed for slide preparation. The slides were stained with Giemsa stain (5% v/v). The slides were evaluated for the Mitotic Index (% of cells in metaphase).

### Mammalian bone marrow erythrocyte micronucleus assay

An in vivo mammalian bone marrow erythrocyte micronucleus assay was conducted in accordance with the OECD Guideline 474 [23]. The micronucleus test was conducted at doses of 500, 1,000 and 2,000 mg/kg. The dose levels for the micronucleus test were selected based on a dose range-finding study. In the micronucleus test, HemoHIM was orally administered to Swiss albino mice (five animals/sex/group) at a dose volume of 10 mL/kg for 2 days, with an interval of approximately 24 h. Animals in the positive control group received a single dose of cyclophosphamide monohydrate intraperitoneally at 40 mg/kg a day before bone marrow collection. Approximately 24 h after dosing, all animals were euthanized and both femur bones were collected from each animal. The bone marrow was collected using 100% FBS. After collection, all samples were centrifuged and the supernatant was discarded, leaving a small amount of an FBS cell pellet. Smears were prepared on slides using the cell pellet. The slides were air-dried, fixed with absolute methanol, and stained with 5% Giemsa stain.

### Statistical method

The study data was tabulated in MS Excel and subjected to statistical analysis using Graph Pad Prism Software. The data of body weight, weight gain, feed consumption, organ weight (absolute and relative), hematological and clinical chemistry estimations, were subjected to statistical analysis. The data is checked for normality and homogeneity prior to statistical comparisons. All the normal and homogenous data is analyzed using One way ANOVA followed by Dunnett’s multiple comparisons in main groups and Student’s *t*-test in recovery groups whereas non-normal and/or non-homogenous data is analyzed using Kruskal-Walis test followed by Dunn’s multiple comparisons in main groups and Mann–Whitney *U*-Test in recovery groups respectively. All analysis and comparisons were evaluated at the 95% level of confidence (*p* < 0.05).

## Results

### Acute oral toxicity

The acute oral toxicity of HemoHIM was investigated in Sprague–Dawley rats according to the OECD Test guideline 423. Initially, three animals (Set-I) of each sex were administered a starting dose of 300 mg/kg and observed for mortality or morbidity. If the animals survived, a confirmatory test was performed using three additional animals (Set-II) of each sex at 300 mg/kg. If these animals survived at a dose of 300 mg/kg, an additional three male and three female animals (Set-III) were administered 2000 mg/kg and observed for mortality or morbidity. If the animals survived, three more male and female rats (Set-IV) were administered the same dose level of 2,000 mg/kg/day (Table 1).

**Table 1:** Experimental design for acute oral toxicity

Set	Dose (mg/kg)	Animal number	
		Male	Female
Set–I	300	1–3	4–6
Set–II		7–9	10–12
Set–III	2000	13–15	16–18
Set–IV		19–21	22–24

No morbidity/mortality or clinical signs of toxicity were observed in any of the animals throughout the experimental period for up to 2,000 mg/kg/day, the limit dose of OECD TG 423 (data not shown). Normal body weight gain was observed in all the animals. A gross pathology examination revealed no abnormalities (data not shown).

### 28-Day repeated dose oral toxicity

A repeated-dose 28-day oral toxicity study was conducted in accordance with the OECD Guideline 407 [19]. HemoHIM was administered orally to Sprague–Dawley rats (five rats/sex/group) at graduated dose levels of 500 mg/kg/day for low-dose (G2), 1,000 mg/kg/day for mid-dose (G3), 2,000 mg/kg/day for high-dose (G4), and high-dose recovery (G4R) groups for 28 consecutive days, followed by a 14-day recovery period. The vehicle or HemoHIM was administered to each group of rats once daily for 28 consecutive days. After 28 days of treatment, the administration of vehicle and test item dose preparation to the control recovery (G1R) and high-dose recovery (G4R) groups was discontinued, and the potential reversibility or persistence of any toxic effects was observed for 14 days.

#### Mortality, clinical observations, and gross pathology

All animals survived until euthanasia. No test item-related clinical signs were observed at any of the doses tested in either sex. Additionally, no test item-related findings were noted in the gross pathology performed at the end of the treatment and recovery periods.

#### Body weights and feed consumption

**Fig. 1:**
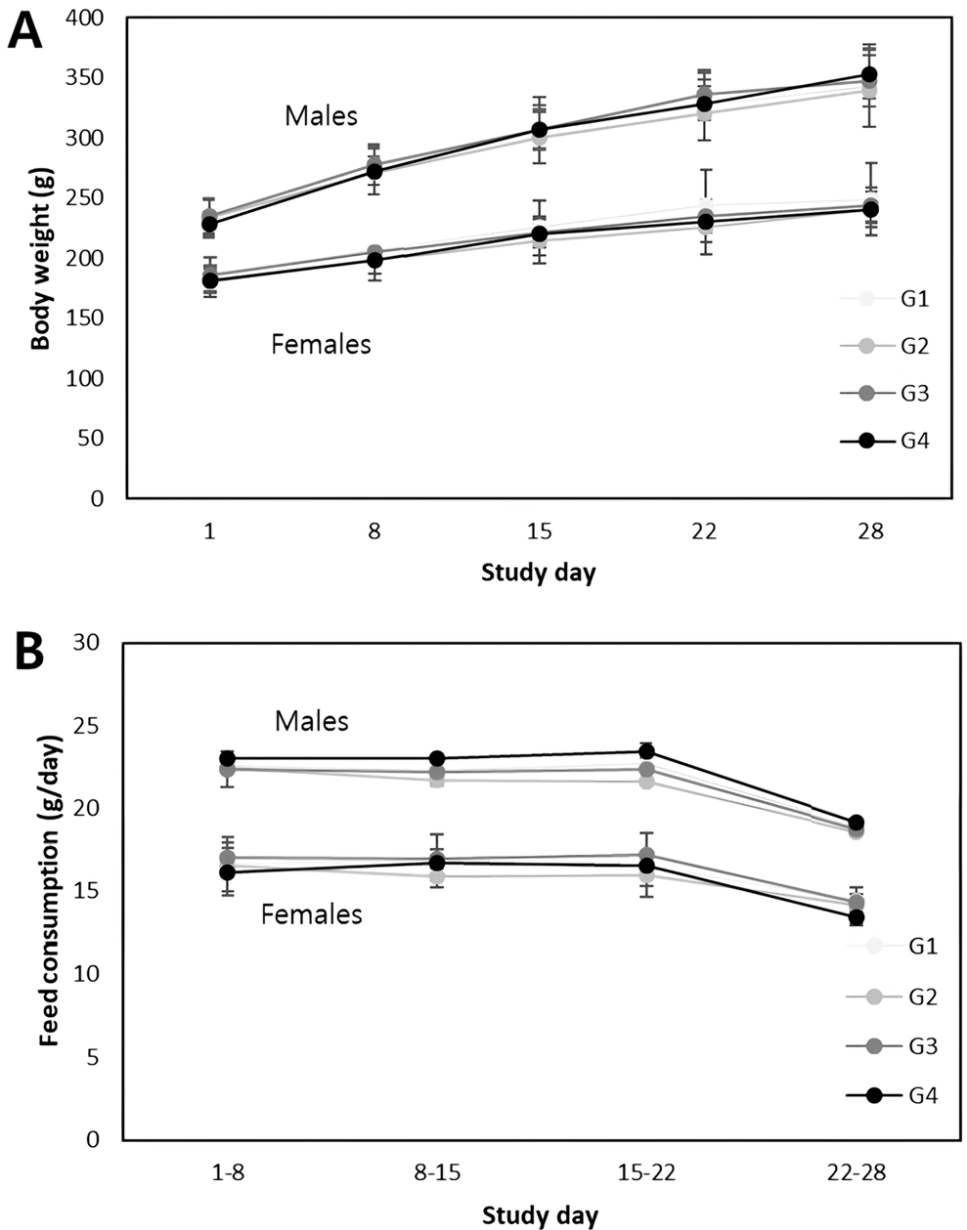
**a** Mean body weights, and **b** mean feed consumption. HemoHIM was administered to rats at graduated dose levels of 0 mg/kg/day (G1), 500 mg/kg/day (G2), 1000 mg/kg/day (G3), and 2000 mg/kg/day (G4)

No test items related to changes in body weight or body weight gain were observed in either sex at any of the doses tested throughout the observation period (Fig. 1A). Normal feed consumption was observed in both sexes at all tested doses during the observation and recovery periods (Fig. 1B).

#### Hematology and clinical chemistry

There were no adverse effects on hematological or clinical chemistry parameters in the high-dose group (G4) compared to the control group (G1) (Tables 2 and 3). Additionally, no significant changes were observed following the recovery periods (data not shown).

**Table 2:** Hematology values for male and female rats

	Males				Females			
	G1	G2	G3	G4	G1	G2	G3	G4
RBC (10^6^ cells/μL)	8.7 ± 0.2	8.7 ± 0.3	9.0 ± 0.4	8.6 ± 0.2	8.1 ± 0.3	8.3 ± 0.4	8.4 ± 0.6	8.1 ± 0.6
Hb (g/dL)	17.0 ± 0.4	16.9 ± 0.1	17.0 ± 0.6	16.9 ± 0.3	16.3 ± 0.2	16.4 ± 0.3	16.3 ± 0.3	16.2 ± 0.9
Hct (%)	47.5 ± 1.3	48.4 ± 1.6	49.2 ± 2.1	48.0 ± 0.9	45.8 ± 0.7	46.4 ± 1.1	46.4 ± 1.6	45.2 ± 2.8
MCV (fL)	54.6 ± 0.8	55.4 ± 0.7	54.9 ± 1.1	55.5 ± 0.7	56.4 ± 1.3	56.1 ± 2.1	55.5 ± 1.8	55.6 ± 1.1
MCH (pg)	19.5 ± 0.2	19.4 ± 0.4	19.0 ± 0.4	19.6 ± 0.2	20.0 ± 0.5	19.8 ± 0.7	19.5 ± 1.0	19.9 ± 0.5
MCHC (g/dL)	35.7 ± 0.7	35.0 ± 1.0	34.6 ± 0.5	35.2 ± 0.2	35.5 ± 0.3	35.3 ± 0.4	35.1 ± 0.8	35.8 ± 0.4
Plt (10^3^ cells/μL)	1116 ± 142	1061 ± 77	995 ± 187	1084 ± 169	1132 ± 170	987 ± 86	1119 ± 14	1097 ± 92
WBC (10^3^ cells/μL)	10.6 ± 2.6	9.5 ± 3.8	11.3 ± 1.3	9.3 ± 2.6	8.0 ± 1.3	7.5 ± 1.4	7.3 ± 1.3	9.2 ± 3.3
Neu (%)	18.1 ± 1.6	17.1 ± 1.5	17.4 ± 2.3	18.1 ± 3.2	18.4 ± 3.0	16.1 ± 1.3	18.1 ± 3.1	17.8 ± 4.1
Lym (%)	80.5 ± 1.6	81.0 ± 1.4	80.9 ± 2.1	80.1 ± 3.5	79.4 ± 3.2	81.6 ± 2.0	79.3 ± 3.4	80.1 ± 5.6
Mono (%)	1.2 ± 0.2	1.5 ± 0.6	1.3 ± 0.7	1.4 ± 0.5	1.7 ± 0.7	1.7 ± 1.3	2.0 ± 1.2	1.6 ± 1.7
Eoso (%)	0.3 ± 0.1	0.4 ± 0.2	0.4 ± 0.1	0.5 ± 0.3	0.5 ± 0.3	0.6 ± 0.1	0.6 ± 0.3	0.6 ± 0.3
Baso (%)	0.0 ± 0.0	0.0 ± 0.0	0.2 ± 0.4	0.0 ± 0.0	0.0 ± 0.0	0.0 ± 0.0	0.0 ± 0.0	0.0 ± 0.0
Rec (%)	2.2 ± 0.2	2.3 ± 0.1	2.3 ± 0.2	2.2 ± 0.2	2.3 ± 0.1	2.2 ± 0.2	2.1 ± 0.2	2.2 ± 0.2
CT (Sec)	102 ± 16	102 ± 16	108 ± 16	96 ± 13	96 ± 13	96 ± 13	102 ± 16	108 ± 16

Each value is presented as the mean ± SD (n = 5)

**Table 3:** Clinical chemistry values for male and female rats

	Males				Females			
	G1	G2	G3	G4	G1	G2	G3	G4
ALB (g/dL)	3.8 ± 0.1	3.8 ± 0.1	3.7 ± 0.1	3.7 ± 0.1	3.8 ± 0.2	3.8 ± 0.1	3.7 ± 0.2	3.7 ± 0.1
ALP (U/L)	285.6 ± 34.3	287.6 ± 26.2	273.4 ± 28.6	288.6 ± 19.5	143.4 ± 11.6	139.4 ± 15.4	147.4 ± 31.2	145.6 ± 13.0
ALT (U/L)	51.8 ± 4.3	55.0 ± 17.0	54.2 ± 6.1	47.2 ± 7.8	39.2 ± 9.6	47.0 ± 6.7	39.2 ± 7.3	40.0 ± 1.9
AST (U/L)	164.2 ± 17.1	160.0 ± 56.4	173.0 ± 42.7	153.0 ± 35.6	134.4 ± 13.2	139.6 ± 25.1	130.0 ± 27.2	127.6 ± 17.5
T.Bil (mg/dL)	0.1 ± 0.0	0.1 ± 0.0	0.1 ± 0.0	0.1 ± 0.0	0.1 ± 0.0	0.1 ± 0.0	0.1 ± 0.0	0.1 ± 0.0
BUN (mg/dL)	18.4 ± 3.8	18.8 ± 1.1	16.4 ± 1.8	19.6 ± 1.9	15.6 ± 3.4	17.0 ± 0.7	16.6 ± 3.3	19.0 ± 2.3
Ca (mg/dL)	10.5 ± 0.2	10.5 ± 0.2	10.2 ± 0.3	10.3 ± 0.7	10.4 ± 0.2	10.7 ± 0.2	10.6 ± 0.4	10.3 ± 0.2
T.Chol (mg/dL)	88.4 ± 4.3	88.6 ± 7.1	87.4 ± 9.9	82.0 ± 10.7	96.4 ± 8.6	86.2 ± 14.9	86.0 ± 10.8	102.2 ± 13.8
Crea (mg/dL)	0.4 ± 0.1	0.4 ± 0.1	0.5 ± 0.1	0.4 ± 0.1	0.4 ± 0.1	0.4 ± 0.1	0.5 ± 0.1	0.5 ± 0.1
Glu (mg/dL)	115.8 ± 16.1	110.4 ± 12.7	120.6 ± 18.0	123.2 ± 19	121.8 ± 9.8	121.4 ± 13.1	111.8 ± 11.0	118.4 ± 9.6
Phos (mg/dL)	8.5 ± 0.4	8.6 ± 0.5	7.9 ± 0.5	8.2 ± 0.9	7.1 ± 0.2	6.8 ± 0.5	7.0 ± 0.6	6.8 ± 0.6
TP (g/dL)	7.0 ± 0.1	7.0 ± 0.2	6.9 ± 0.3	6.8 ± 0.3	6.7 ± 0.2	6.9 ± 0.3	6.8 ± 0.4	6.7 ± 0.2
Trig (mg/dL)	66.0 ± 16.9	64.8 ± 13.2	73.2 ± 16.7	74.0 ± 14.8	65.2 ± 15.1	74.4 ± 16.7	70.0 ± 21.2	70.4 ± 15.5
LDL (mg/dL)	17.0 ± 2.1	20.0 ± 1.0	23.0 ± 6.0	19.4 ± 4.2	15.2 ± 1.8	14.0 ± 1.2	14.2 ± 2.9	15.8 ± 3.0
HDL (mg/dL)	40.4 ± 9.3	43.0 ± 5.5	42.6 ± 7.3	44.4 ± 8.8	49.4 ± 7.3	57.6 ± 10.2	61.6 ± 10.1	62.2 ± 8.3
Urea (mg/dL)	39.4 ± 7.5	40.2 ± 1.9	35.2 ± 4.3	41.8 ± 4.5	33.6 ± 7.4	36.4 ± 1.1	36.2 ± 7.0	40.8 ± 4.9
Na (mmol/L)	136.2 ± 0.4	136.2 ± 1.0	136.4 ± 0.9	135.8 ± 0.7	136.4 ± 0.7	136.0 ± 1.2	136.0 ± 1.0	136.3 ± 1.3
K (mmol/L)	4.7 ± 0.1	4.7 ± 0.1	4.7 ± 0.1	4.7 ± 0.2	4.3 ± 0.2	4.2 ± 0.3	4.2 ± 0.1	4.5 ± 0.2
Cl (mmol/L)	97.6 ± 1.3	97.7 ± 0.8	98.3 ± 1.1	97.6 ± 0.9	98.5 ± 0.7	98.7 ± 0.8	98.8 ± 0.6	99.6 ± 1.5

Each value is presented as the mean ± SD (n = 5)

#### Organ weights and histopathology

No biologically significant differences were observed in organ weights or relative organ weights in either sex when compared to the respective vehicle control groups (Tables 4 and 5). There were no test item-related histopathological findings in any organ of the terminally sacrificed male and female rats in the high-dose group (G4) compared to the control group (G1) (Table 6). There were also no treatment related changes was observed following the recovery periods (data not shown).

**Table 4:** Relative organ weight (%) for male rats

	G1	G2	G3	G4
Fasting Body Weight (g)	331.77 ± 35.62	327.52 ± 13.94	341.05 ± 22.9	341.73 ± 20.53
Liver	4.1796 ± 0.4338	4.0411 ± 0.2909	3.9159 ± 0.3937	4.1507 ± 0.0611
Kidneys	0.8699 ± 0.0621	0.7837 ± 0.0710	0.8035 ± 0.0562	0.8293 ± 0.0485
Adrenal Glands	0.0186 ± 0.0044	0.0181 ± 0.0047	0.0179 ± 0.0041	0.0160 ± 0.0020
Heart	0.4115 ± 0.0338	0.3996 ± 0.0310	0.4169 ± 0.0231	0.4063 ± 0.0151
Brain	0.6300 ± 0.0628	0.5944 ± 0.0153	0.5658 ± 0.0735	0.5843 ± 0.0630
Spleen	0.2348 ± 0.0289	0.2225 ± 0.0175	0.2994 ± 0.1254	0.2217 ± 0.0193
Thymus	0.2321 ± 0.0380	0.2132 ± 0.0575	0.1964 ± 0.0337	0.1958 ± 0.0130
Testes	0.9764 ± 0.0728	1.0380 ± 0.0663	0.9633 ± 0.1129	0.9486 ± 0.0923
Epididymides	0.3369 ± 0.0173	0.3163 ± 0.0366	0.3117 ± 0.0408	0.2915 ± 0.0446
SV-CG and Prostate Gland	0.6859 ± 0.0719	0.6154 ± 0.0286	0.6348 ± 0.1025	0.6027 ± 0.0513
Thyroid with Parathyroid Gland	0.0079 ± 0.0011	0.0074 ± 0.0009	0.0082 ± 0.0014	0.0069 ± 0.0007
Pituitary Gland	0.0038 ± 0.0003	0.0042 ± 0.0002	0.0039 ± 0.0001	0.0040 ± 0.0006

Each value is presented as the mean ± SD (n = 5)

**Table 5:** Relative organ weight (%) for female rats

	G1	G2	G3	G4
Fasting Body Weight (g)	244.19 ± 27.95	230.02 ± 21.77	236.91 ± 16.82	234.48 ± 12.54
Liver	3.9311 ± 0.3656	3.5851 ± 0.1810	3.7354 ± 0.1981	3.9853 ± 0.2034
Kidneys	0.7569 ± 0.0323	0.6969 ± 0.0286	0.7654 ± 0.1217	0.7096 ± 0.0831
Adrenal Glands	0.0305 ± 0.0066	0.0320 ± 0.0098	0.0329 ± 0.0057	0.0314 ± 0.0047
Heart	0.4124 ± 0.0344	0.5168 ± 0.1675	0.4377 ± 0.0344	0.4557 ± 0.0562
Brain	0.8084 ± 0.1061	0.8192 ± 0.0562	0.7801 ± 0.0587	0.8454 ± 0.0723
Spleen	0.2879 ± 0.0581	0.2753 ± 0.0259	0.2752 ± 0.0191	0.3120 ± 0.0806
Thymus	0.2265 ± 0.0354	0.2246 ± 0.0599	0.2003 ± 0.0243	0.2920 ± 0.0623
Uterus with CrV	0.2629 ± 0.0732	0.2698 ± 0.1347	0.2657 ± 0.0910	0.2627 ± 0.1104
Ovaries	0.0735 ± 0.0175	0.0655 ± 0.0173	0.0597 ± 0.0115	0.0673 ± 0.0093
Thyroid with Parathyroid Gland	0.0112 ± 0.0013	0.0101 ± 0.0022	0.0098 ± 0.0020	0.0094 ± 0.0012
Pituitary Gland	0.0062 ± 0.0016	0.0061 ± 0.009	0.0058 ± 0.0007	0.0070 ± 0.0009

Each value is presented as the mean ± SD (n = 5)

**Table 6:** Summary of histopathological findings of tissues from the control (G1) and 2,000 mg/kg /day (G4) groups

Organs/ Lesions	Severity	G1		G4	
		Male	Female	Male	Female
Adrenal glands					
Accessory adrenocortical tissue	Not graded	1/5	0/5	0/5	0/5
Clear cells focus	Minimal	0/5	1/5	0/5	0/5
Kidneys					
Tubular basophilia with MNC infiltrate	Minimal	1/5	0/5	0/5	0/5
Tubular basophilia, cortex	Minimal	1/5	0/5	1/5	0/5
Pancreas					
Atrophy	Minimal	0/5	1/5	0/5	0/5
Pituitary gland					
Rathke’s cleft	Not graded	0/5	1/5	0/5	1/5
Thyroids					
Ultimobranchial cyst	Not graded	0/5	1/5	0/5	0/5

Group/incidence of findings (number of animals showing findings of the total number of animals in the group, n = 5)

### 13-Week repeated dose oral toxicity

A repeated-dose 13-week oral toxicity study was conducted in accordance with the OECD Guideline 408 [20]. HemoHIM was administered orally to Sprague–Dawley rats (10 rats/sex/group) at graduated dose levels of 500 mg/kg/day for low-dose (G2), 1,000 mg/kg/day for mid-dose (G3), and 2,000 mg/kg/day for high-dose (G4) groups for 13 consecutive weeks. The vehicle or HemoHIM was administered to each group once daily for 13 consecutive weeks. During the observation period, clinical and detailed clinical signs, measurement of body weight and food consumption, ophthalmological examinations, and urinalysis were observed. At the end of the observation period, hematological, clinical chemistry, and histopathological examinations were performed.

#### Mortality, clinical observations, and gross pathology

Throughout the dosing period, no test item-related death and clinical sings were observed at any of the doses tested in either sex. Detailed examination of clinical signs revealed no abnormal changes in either the control or HemoHIM-treated groups. Furthermore, no abnormalities were detected in ophthalmological examinations of the surviving animals (data not shown).

#### Body weights and feed consumption

**Fig. 2:**
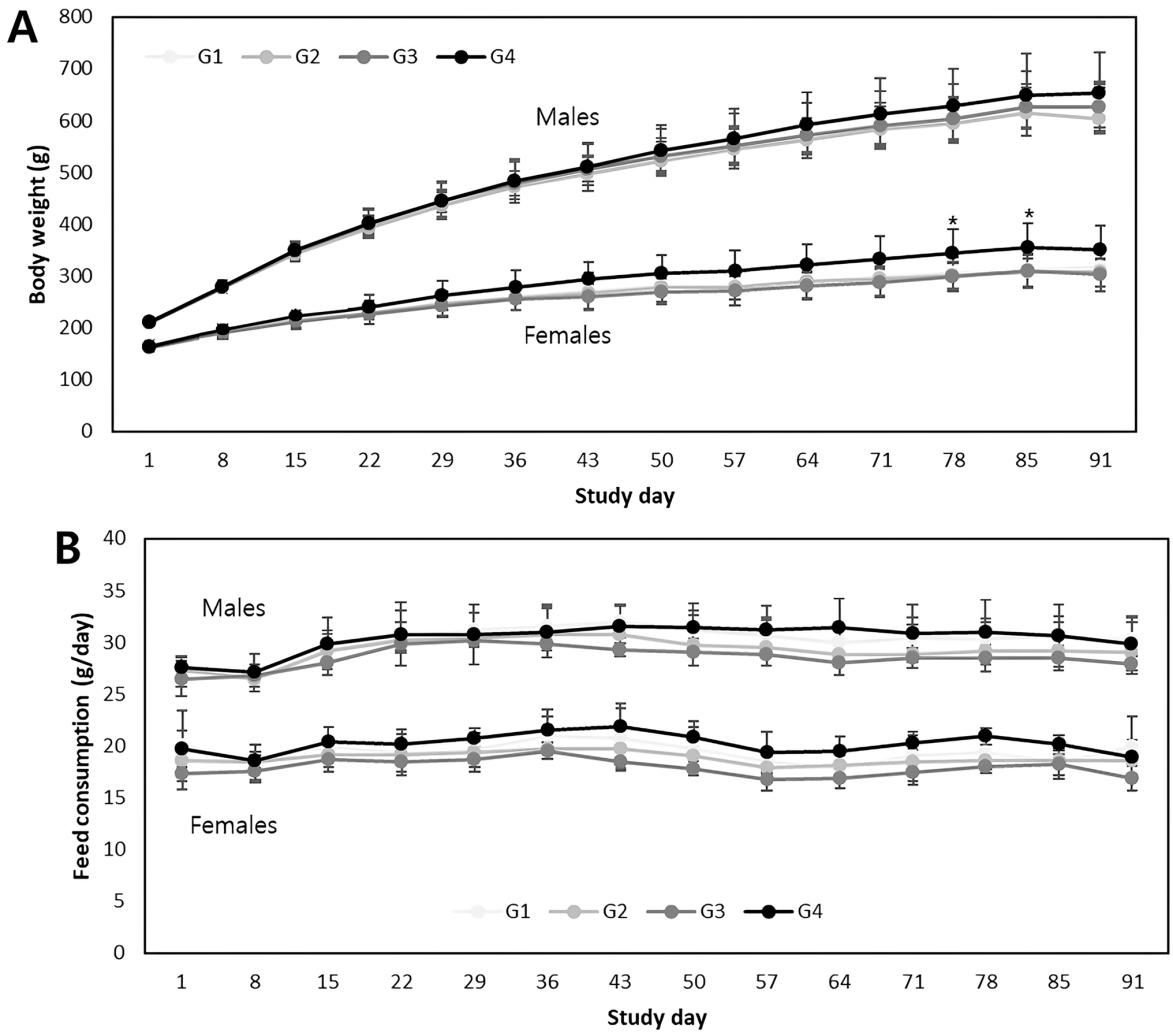
**a** Mean body weights, and** b** mean feed consumption. HemoHIM was administered to rats at graduated dose levels, including a control group (G1) receiving 0 mg/kg/day, and treatment groups (G2–4) receiving doses of 500, 1000, and 2000 mg/kg/day. Significant differences were determined using Dunnett’s *t*-test: *p < 0.05, **p < 0.01

Throughout the dosing period, no significant toxicological changes in body weight were observed in the HemoHIM-treated groups of either sex. However, in the 2,000 mg/kg/day dosing group of female animals, a slightly higher body weight was noted on days 57–91 compared to that in the control group, with an increase of up to 13.8%. Although these changes were considered to be related to the test items owing to their gradual and consistent nature during the dosing period, they were not accompanied by any abnormal clinical signs or hematological or morphological changes and were thus deemed non-toxicological. Similarly, the observed differences in body weight in the other dosing groups were deemed unrelated to the test items because of their small magnitudes (<10%) or lack of persistence (Fig. 2A). Furthermore, there were no test item-related changes in feed consumption in HemoHIM- treated groups of either sex during the dosing period (Fig. 2B).

#### Urinalysis, hematology and clinical chemistry

During the dosing period, no significant toxicological changes were observed in the urinalysis of HemoHIM-treated groups of either sex (data not shown). Hematological and clinical parameters did not show any significant toxicological changes in the HemoHIM-treated groups of either sex. Sporadic differences in other parameters, even if statistically significant, were not considered to be related to the test item because of the absence of a dose–response relationship and correlation with other parameters (Tables 7 and 8).

**Table 7:** Hematology values for male and female rats

	Males				Females			
	G1	G2	G3	G4	G1	G2	G3	G4
RBC (10^6^ cells/μL)	8.27 ± 0.24	8.41 ± 0.42	8.24 ± 0.20	8.22 ± 0.47	7.66 ± 0.33	7.34 ± 0.31	7.36 ± 0.39	7.46 ± 0.26
Hb (g/dL)	15.1 ± 0.6	14.9 ± 0.6	15.2 ± 0.5	15.0 ± 0.5	14.4 ± 0.4	14.0 ± 0.6	14.1 ± 0.6	14.1 ± 0.4
Hct (%)	43.0 ± 1.5	42.8 ± 1.5	43.2 ± 1.0	42.8 ± 1.4	40.9 ± 0.8	39.9 ± 1.8	40.2 ± 1.7	40.3 ± 1.2
MCV (fL)	52.1 ± 1.7	51.0 ± 1.0	52.4 ± 1.2	52.2 ± 2.0	53.4 ± 1.7	54.3 ± 1.3	54.6 ± 1.6	54.1 ± 1.5
MCH (pg)	18.3 ± 0.8	17.7 ± 0.4	18.4 ± 0.5	18.3 ± 0.9	18.9 ± 0.5	19.1 ± 0.4	19.2 ± 0.6	18.9 ± 0.5
MCHC (g/dL)	35.1 ± 0.6	34.7 ± 0.4	35.2 ± 0.4	35.0 ± 0.5	35.4 ± 0.5	35.1 ± 0.6	35.1 ± 0.5	34.9 ± 0.3
Plt (10^3^ cells/μL)	1072 ± 98	1076 ± 101	964 ± 67*	1017 ± 95	981 ± 106	988 ± 74	1005 ± 110	971 ± 112
Rec (%)	3.29 ± 0.40	3.38 ± 0.63	3.42 ± 0.58	3.56 ± 0.41	3.17 ± 0.91	3.20 ± 0.61	3.66 ± 1.40	3.53 ± 0.45
WBC (10^3^ cells/μL)	11.2 ± 2.3	11.2 ± 1.5	11.0 ± 2.7	10.0 ± 1.5	5.5 ± 1.0	5.9 ± 2.4	5.4 ± 1.0	5.8 ± 1.2
Neu (%)	18.3 ± 5.2	20.1 ± 6.6	18.7 ± 5.1	20.0 ± 8.5	13.1 ± 2.2	19.1 ± 5.5	15.8 ± 6.1	16.3 ± 4.2
Lym (%)	71.3 ± 6.6	69.4 ± 7.6	70.7 ± 5.2	68.4 ± 9.0	76.0 ± 2.3	68.5 ± 5.3*	73.6 ± 7.5	72.1 ± 5.7
Mono (%)	9.1 ± 1.9	9.1 ± 1.8	9.2 ± 1.5	10.2 ± 1.6	9.1 ± 1.5	10.9 ± 2.3	9.1 ± 1.7	9.9 ± 2.1
Eoso (%)	1.0 ± 0.2	1.1 ± 0.4	1.0 ± 0.5	1.0 ± 0.3	1.5 ± 0.8	1.2 ± 0.3	1.3 ± 0.4	1.4 ± 0.5
Baso (%)	0.4 ± 0.1	0.4 ± 0.1	0.4 ± 0.1	0.4 ± 0.1	0.4 ± 0.1	0.3 ± 0.1	0.2 ± 0.1	0.4 ± 0.1
PT (Sec)	18.5 ± 0.4	17.6 ± 0.6*	18.1 ± 0.6	18.2 ± 0.8	17.9 ± 0.9	17.4 ± 0.7	17.2 ± 0.7	17.7 ± 0.8
APTT (Sec)	16.0 ± 1.3	16.2 ± 0.7	15.4 ± 1.5	16.0 ± 1.4	14.4 ± 1.1	14.2 ± 1.9	13.3 ± 1.7	13.5 ± 1.4

Each value is presented as the mean ± SD. Significant differences were determined using Dunnett’s *t*-test: ^*^p < 0.05, ^**^p < 0.01

**Table 8:** Clinical chemistry values for male and female rats

	Males				Females			
	G1	G2	G3	G4	G1	G2	G3	G4
ALT (U/L)	26.8 ± 4.2	25.1 ± 2.0	24.7 ± 5.1	24.0 ± 2.9	30.4 ± 23.5	24.5 ± 4.6	47.5 ± 53.2	25.6 ± 6.3
AST (U/L)	85.1 ± 20.0	69.6 ± 16.9	72.2 ± 11.8	74.3 ± 15.3	90.1 ± 52.5	78.4 ± 20.1	138.8 ± 169.5	74.7 ± 8.5
ALP (U/L)	227.6 ± 28.9	219.1 ± 35.7	221.5 ± 33.1	214.4 ± 25.0	137.2 ± 62.2	120.3 ± 52.9	111.8 ± 35.7	113.4 ± 23.7
GGT (U/L)	0.05 ± 0.09	0.05 ± 0.07	0.04 ± 0.11	0.03 ± 0.06	0.13 ± 0.18	0.15 ± 0.23	0.15 ± 0.15	0.04 ± 0.09
Glu (mg/dL)	136 ± 11	145 ± 15	140 ± 15	139 ± 12	135 ± 14	136 ± 13	137 ± 9	140 ± 10
BUN (mg/dL)	13.5 ± 1.5	13.6 ± 1.6	13.2 ± 1.3	12.9 ± 1.1	15.0 ± 2.0	15.1 ± 1.6	15.0 ± 3.2	14.8 ± 2.0
Crea (mg/dL)	0.47 ± 0.05	0.50 ± 0.03	0.50 ± 0.03	0.46 ± 0.04	0.57 ± 0.06	0.58 ± 0.05	0.60 ± 0.06	0.59 ± 0.06
T.Bili (mg/dL)	0.07 ± 0.02	0.05 ± 0.01	0.06 ± 0.02	0.07 ± 0.02	0.09 ± 0.02	0.08 ± 0.02	0.09 ± 0.03	0.09 ± 0.03
T.Chol (mg/dL)	68 ± 10	75 ± 9	75 ± 13	75 ± 23	84 ± 29	91 ± 16	89 ± 16	88 ± 21
Trig (mg/dL)	42 ± 26	45 ± 9	45 ± 11	77 ± 44	37 ± 34	38 ± 46	36 ± 19	59 ± 40
TP (g/dL)	5.7 ± 0.2	5.8 ± 0.2	6.0 ± 0.2	5.9 ± 0.3	6.5 ± 0.6	6.7 ± 0.5	6.8 ± 0.5	6.8 ± 0.4
ALB (g/dL)	2.3 ± 0.1	2.3 ± 0.1	2.4 ± 0.1	2.4 ± 0.1*	3.0 ± 0.4	3.1 ± 0.3	3.2 ± 0.3	3.1 ± 0.2
A/G ratio	0.66 ± 0.03	0.65 ± 0.04	0.66 ± 0.05	0.67 ± 0.02	0.85 ± 0.08	0.83 ± 0.05	0.86 ± 0.07	0.83 ± 0.03
Phos (mg/dL)	6.13 ± 0.30	6.28 ± 0.52	6.42 ± 0.35	6.47 ± 0.17	4.60 ± 0.62	4.34 ± 0.43	4.46 ± 0.53	4.58 ± 0.72
Ca (mg/dL)	9.4 ± 0.2	9.3 ± 0.2	9.6 ± 0.3	9.5 ± 0.2	9.2 ± 0.6	9.4 ± 0.3	9.5 ± 0.3	9.4 ± 0.3
Na (mmol/L)	142.7 ± 0.9	142.3 ± 1.3	142.6 ± 0.9	142.7 ± 0.8	141.0 ± 0.8	140.3 ± 0.9	141.0 ± 0.5	140.2 ± 0.8*
K (mmol/L)	4.31 ± 0.29	4.43 ± 0.29	4.44 ± 0.21	4.56 ± 0.22	3.93 ± 0.26	3.94 ± 0.27	4.01 ± 0.28	3.96 ± 0.30
Cl (mmol/L)	103.1 ± 1.1	103.0 ± 0.7	102.8 ± 1.3	102.5 ± 1.1	104.1 ± 1.7	104.2 ± 2.0	103.7 ± 1.1	102.5 ± 1.7
TBA (mmol/L)	14.9 ± 7.8	11.1 ± 9.7	11.2 ± 6.5	11.5 ± 8.0	14.3 ± 13.2	11.6 ± 5.2	19.1 ± 18.7	12.6 ± 6.2
Urea (mg/dL)	29 ± 3	29 ± 3	28 ± 3	28 ± 2	32 ± 4	32 ± 3	32 ± 7	32 ± 4
HDL (mg/dL)	18.1 ± 2.0	19.5 ± 1.8	20.2 ± 2.3	20.0 ± 3.7	25.2 ± 6.9	26.9 ± 3.8	26.8 ± 4.3	27.2 ± 4.8
LDL (mg/dL)	5.2 ± 1.5	6.1 ± 1.2	5.6 ± 1.6	5.1 ± 1.8	3.7 ± 1.1	3.6 ± 0.6	3.9 ± 0.6	3.6 ± 0.8
T4 (ng/dL)	62.4 ± 5.4	63.3 ± 9.9	61.4 ± 12.6	66.0 ± 8.9	33.5 ± 9.4	32.2 ± 8.9	32.9 ± 8.8	32.0 ± 9.8
T3 (ng/dL)	0.990 ± 0.086	0.925 ± 0.117	0.921 ± 0.119	1.048 ± 0.115	0.781 ± 0.107	0.848 ± 0.136	0.859 ± 0.127	0.725 ± 0.124
TSH (ng/dL)	2.62 ± 1.18	4.68 ± 2.03*	5.92 ± 1.34**	4.13 ± 2.39	3.25 ± 1.64	3.21 ± 1.89	4.73 ± 2.47	5.89 ± 3.17*

Each value is presented as the mean ± SD. Significant differences were determined using Dunnett’s *t*-test: ^*^p < 0.05, ^**^p < 0.01

#### Organ weights and histopathology

There were no significant toxicological changes in organ weight in the HemoHIM-treated groups of either sex. Statistically significant differences were observed in the mean relative organ weights of the liver in males and kidneys of females between the control and HemoHIM-treated groups. However, these differences were not considered to be related to the test item because there was no dose–response relationship, and no correlated morphological findings were observed (Tables 9 and 10). Macroscopic and microscopic examinations did not reveal any test item-related changes in any of the animals. All gross and microscopic findings observed were isolated incidences and considered incidental or congenital changes without toxicological significance. Upon examination, all lobes of the lungs were dilated and filled with fluid, and the lung weight increased. However, this was determined to be the result of accidental inflow of foreign materials (regardless of lung inflation using formalin) into the lungs and was not related to the test item (data not shown). Additionally, regarding the results of observation of the estrus cycle, compared to the histopathological examination of the female genital organs, there were no significant changes (data not shown). Therefore, the NOAEL (No Observable Adverse Effect Level) of the test substance, HemoHIM, was considered to be at 2,000 mg/kg/day for both sexes of rats.

**Table 9:** Relative organ weights (%) of male rats

	G1	G2	G3	G4
Fasting Body Weight (g)	601.1 ± 43.8	577.3 ± 65.5	600.0 ± 34.7	625.6 ± 75.4
Adrenal glands	0.0110 ± 0.0018	0.0098 ± 0.0021	0.0105 ± 0.0020	0.0101 ± 0.0016
Brain	0.3793 ± 0.0027	0.3842 ± 0.0346	0.3718 ± 0.0216	0.3672 ± 0.0350
Epididymis	0.2814 ± 0.0306	0.2837 ± 0.0300	0.2736 ± 0.0253	0.2643 ± 0.0204
Heart	0.2702 ± 0.0144	0.2687 ± 0.0153	0.2684 ± 0.0236	0.2626 ± 0.0186
Kidneys	0.5856 ± 0.0372	0.5850 ± 0.0410	0.5707 ± 0.0443	0.5941 ± 0.0340
Liver	2.4433 ± 0.1678	2.6377 ± 0.1520*	2.5351 ± 0.2008	2.6476 ± 0.1103*
Pituitary gland	0.0024 ± 0.0003	0.0026 ± 0.0004	0.0025 ± 0.0004	0.0024 ± 0.0004
SV-CG and Prostate Gland	0.6509 ± 0.0587	0.6871 ± 0.0958	0.6011 ± 0.0532	0.6368 ± 0.0664
Spleen	0.1628 ± 0.0190	0.1676 ± 0.0077	0.1691 ± 0.0286	0.1598 ± 0.0220
Testis	0.6870 ± 0.0490	0.6748 ± 0.0727	0.6696 ± 0.0500	0.6318 ± 0.0664
Thymus	0.0670 ± 0.0156	0.0578 ± 0.0117	0.0613 ± 0.0154	0.0596 ± 0.0112
Thyroid gland with parathyroid gland	0.0040 ± 0.0007	0.0045 ± 0.0009	0.0044 ± 0.0007	0.0040 ± 0.0006

Each value is presented as the mean ± SD. Significant differences were determined using Dunnett’s *t*-test: ^*^p < 0.05, ^**^p < 0.01

**Table 10:** Relative organ weights (%) of female rats

	G1	G2	G3	G4
Fasting Body Weight (g)	306.5 ± 33.4	300.6 ± 22.0	296.2 ± 31.4	344.3 ± 45.7*
Adrenal glands	0.0228 ± 0.0047	0.0229 ± 0.0034	0.0231 ± 0.0042	0.0203 ± 0.0044
Brain	0.6608 ± 0.0537	0.6637 ± 0.0465	0.6742 ± 0.0804	0.6027 ± 0.0888
Heart	0.3320 ± 0.0246	0.3304 ± 0.0293	0.3299 ± 0.0221	0.3129 ± 0.0264
Kidneys	0.6569 ± 0.0314	0.6417 ± 0.0551	0.6353 ± 0.0531	0.5898 ± 0.0610*
Liver	2.4974 ± 0.2422	2.5941 ± 0.2795	2.6714 ± 0.2348	2.5409 ± 0.1921
Ovaries	0.0298 ± 0.0082	0.0301 ± 0.0053	0.0288 ± 0.0082	0.0264 ± 0.0058
Pituitary gland	0.0066 ± 0.0016	0.0068 ± 0.0011	0.0069 ± 0.0009	0.0058 ± 0.0014
Spleen	0.2004 ± 0.0264	0.1949 ± 0.0253	0.1814 ± 0.0220	0.1748 ± 0.0244
Thymus	0.1107 ± 0.0270	0.1034 ± 0.0255	0.0937 ± 0.0182	0.0968 ± 0.0203
Thyroid gland with parathyroid gland	0.0069 ± 0.0008	0.0070 ± 0.0008	0.0075 ± 0.0013	0.0071 ± 0.0011
Uterus with CrV	0.2308 ± 0.0637	0.3070 ± 0.1018	0.2319 ± 0.0665	0.2021 ± 0.0476

Each value is presented as the mean ± SD. Significant differences were determined using Dunnett’s *t*-test: ^*^p < 0.05, ^**^p < 0.01

### Bacterial reverse mutation test

An in vitro bacterial reverse mutation assay was conducted using HemoHIM in accordance with the OECD Guideline 471 and in accordance with OECD principles of GLP (as revised 1997) ENV/MC/CHEM(98)17. No precipitation and cytotoxicity in the form of background lawn reduction and revertant count was observed at all the concentrations tested (ranging from 312.5 to 5000.0 μg HemoHIM/plate with (5% v/v, S9) or without metabolic activation, when compared to vehicle control plates. The positive controls responded as expected. The mean number of revertant colonies was within the acceptable range of historical data for the vehicle and positive controls (Tables 11 and 12).

**Table 11:** Mean number of revertants ± SD with metabolic activation

Conc. (μg/plate)	TA1537	TA1535	TA98	TA100	WP2*uvrA*
Vehicle control					
0.0	11.00 ± 1.00	14.67 ± 1.53	22.33 ± 1.53	122.00 ± 2.65	11.33 ± 0.58
HemoHIM					
312.5	10.00 ± 2.00	15.00 ± 2.65	24.00 ± 1.73	121.00 ± 1.73	11.00 ± 2.00
625.0	10.67 ± 1.53	14.33 ± 3.06	23.33 ± 2.52	120.33 ± 2.52	12.33 ± 2.52
1250.0	10.00 ± 1.00	14.67 ± 0.58	25.33 ± 1.53	121.00 ± 2.00	13.33 ± 1.15
2500.0	10.33 ± 2.08	13.67 ± 2.08	23.67 ± 3.51	118.67 ± 2.08	16.00 ± 1.00
5000.0	10.33 ± 0.58	14.67 ± 2.08	25.00 ± .1.00	121.33 ± 1.53	11.00 ± 2.65
Positive control (2-Aminoanthracene)					
20.0	208.67 ± 15.04	1217.33 ± 59.48	1242.67 ± 45.49	1260.33 ± 46.61	–
30.0	–	–	–	–	182.33 ± 8.74

**Table 12:** Mean number of revertants ± SD without metabolic activation

Conc. (μg/plate)		TA1537	TA1535	TA98	TA100	WP2*uvrA*
Vehicle control						
0.0		10.00 ± 1.00	13.33 ± 1.53	24.33 ± 2.08	120.00 ± 2.65	10.00 ± 1.00
HemoHIM						
312.5		10.67 ± 1.53	13.00 ± 1.73	23.67 ± 2.31	121.33 ± 3.51	11.33 ± 3.21
625.0		11.33 ± 1.15	15.00 ± 1.00	25.00 ± 2.65	121.33 ± 2.08	9.00 ± 1.00
1250.0		10.33 ± 1.15	14.00 ± 1.00	24.00 ± 3.00	119.00 ± 2.00	9.67 ± 1.53
2500.0		10.33 ± 1.53	13.33 ± 0.58	23.67 ± 2.52	121.33 ± 1.53	11.00 ± 2.00
5000.0		11.00 ± 1.00	15.00 ± 1.00	23.00 ± 2.00	121.00 ± 2.65	10.33 ± 1.53
Positive control						
9AA	50.0	213.67 ± 11.02	–	–	–	–
SA	10.0	–	1245.67 ± 50.50	–	1234.00 ± 55.57	–
2NF	25.0	–	–	1422.67 ± 112.59	–	–
4NQO	3.0	–	–	–	–	189.33 ± 22.68

*9AA* 9-aminoacridine, *SA* Sodium azide, *2NF* 2-nitrofluorene, *4NQO* 4-nitroquinoline-N-oxide

### In vitro mammalian chromosomal aberration test

An in vitro mammalian chromosomal aberration assay was conducted using HemoHIM in accordance with the OECD Guideline 473 and in accordance with OECD principles of GLP (as revised 1997) ENV/MC/CHEM(98)17. Cytotoxicity (significant reduction in the Mitotic Index) was not observed for any of the tested concentrations under short and continuous exposure conditions. In short-term and continuous exposures, no statistically significant increase in the percentage of cells with structural chromosomal aberrations was observed for any of the tested doses of HemoHIM when compared to the vehicle control. The proportion of structural chromosome-aberrant cells in vehicle control cultures was within the historical range. Treatment with the positive controls (cyclophosphamide monohydrate and ametycin) resulted in a statistically significant increase in the proportion of cells showing structural chromosomal aberrations. The results for the vehicle and positive controls were as expected, confirming the test system sensitivity, S9 mix effectiveness, and assay validity (Table 13).

**Table 13:** Chromosome aberration assay

Conc. (μg/mL)	S9	Exposure-recovery (h)	% of MI	% Reduction in MI	No. of structural chromosome aberrant cells		No. of numerical chromosome aberrant cells
					Incl. gap	Excl. gap	
0 (VC)	−	4–18	9.25	NA	2	2	0
156.25	−	4–18	7.39	20.15	2	2	0
312.5	−	4–18	6.50	29.73	1	1	0
625	−	4–18	5.59	39.60	2	2	0
0.3 (APC)	−	4–18	7.09	23.43	18^*^	17^*^	0
0 (VC)	+	4–18	9.08	NA	2	2	0
156.25	+	4–18	7.42	18.32	1	1	0
312.5	+	4–18	6.38	29.74	2	2	0
625	+	4–18	5.59	38.46	1	1	0
10 (CPPC)	+	4–18	6.88	24.25	18^*^	18^*^	0
0 (VC)	−	22–0	8.78	NA	2	2	0
156.25	−	22–0	7.02	20.07	3	2	0
312.5	−	22–0	5.98	31.91	1	1	0
625	−	22–0	5.28	39.86	2	2	0
0.3 (APC)	−	22–0	6.66	24.12	19^*^	18^*^	0

*MI* mitotic index, *VC* vehicle control, *APC* ametycin positive control, *CPPC* cyclophosphamide monohydrate positive control

^*^Significantly increased compared to vehicle control (*p* < 0.05)

### Mammalian bone marrow erythrocyte micronucleus test

An in vivo mammalian bone marrow erythrocyte micronucleus assay was conducted in accordance with the OECD Guideline 474 and in accordance with OECD principles of GLP (as revised 1997) ENV/MC/CHEM(98)17. None of the animals experienced mortality, morbidity, or clinical signs of toxicity during the study period. The test item did not show any cytotoxicity in the form of a reduced polychromatic erythrocyte (PCE) to total erythrocyte ratio compared with that of the vehicle control. The range of micronucleated PCE observed in the vehicle control animals was 0–3, which was within the laboratory historical range. There was no statistically significant increase in the frequency of micronucleated PCE in animals treated with HemoHIM when compared to the vehicle control. The frequency of micronucleated PCE in animals intraperitoneally treated with cyclophosphamide monohydrate (40 mg/kg) was significantly increased (*p* < 0.001) compared to that of the vehicle control (Table 14).

**Table 14:** Incidence of micronucleated polychromatic erythrocytes in the bone marrow of Swiss albino mice

Concentration (mg/kg)	Mean PCE:TE	% Reduction	% MNPCE (Mean ± SD)
0 (VC)	0.504	NA	0.04 ± 0.02
500	0.502	0.4	0.04 ± 0.02
1,000	0.507	−0.6	0.03 ± 0.02
2,000	0.507	−0.6	0.04 ± 0.02
40 (CPPC)	0.509	−1.0	1.16 ± 0.17^*^

*PCE* polychromatic erythrocyte, *TE* total erythrocyte, *MNPCE* micronucleated polychromatic erythrocyte, *VC* vehicle control, *CPPC* cyclophosphamide monohydrate positive control

^*^Significantly increased compared to vehicle control (*p* < 0.001)

## Discussion

The safety of functional foods is an important aspect since these products are designed to provide additional health benefits beyond basic nutrition. Functional foods are under regulatory supervision for safety in many countries. Guidelines and regulations are mandated to ensure the safety and quality of functional foods in the market. For example, in Korea, safety information such as the rationale for dietary consumption, active ingredients or related substances, daily intake assessment, nutritional evaluation, biological benefits, human test data, and toxicity test data are required for the approval of functional foods.

When assessing the safety of functional foods, reliable intake assessment data from sources such as the National Health and Nutrition Examination Survey should be used. Safety indicators, including hematological/biochemical tests, urine tests, vital signs, and body measurements, should be presented along with adverse reaction cases. Toxicity assessments should follow the OECD Test Guidelines and include tests for acute toxicity, repeated dose toxicity (preferably, 90 days), and genotoxicity. The functional benefits to health should be supported by in vitro and in vivo studies explaining the mechanism of action at a cellular or organismal level [24].

In traditional Oriental medicine, Samul-tang is harnessed to address blood-related ailments such as anemia. This therapeutic approach incorporates ingredients, such as Angelicae gigantis radix, Cnidii rhizoma, Paeoniae radix, and Rehmanniae radix preparata [25, 26]. Previous research has revealed the impact of Samul-tang on hematopoiesis, indicating its influence on various cellular mechanisms within the bone marrow. This encompasses hematopoietic stem cells and blood cell types such as erythrocytes, leukocytes, and thrombocytes. Notably, Samul-tang interacts with crucial hematopoietic factors, including erythropoietin, granulocyte colony-stimulating factor, interleukins, and interferon-gamma [27, 28].

However, when administered at a dosage of 6–12 g per day for 2 months, HemoHIM has a notable impact on immune function in patients, diagnosed with breast or uterine cervix cancer. Moreover, this supplementation is well tolerated by humans because it does not elicit any side effects or display signs of toxicity [29]. Additionally, in a separate study involving healthy adults experiencing fatigue, an 8-week intake of 50% HemoHIM at 40 g per day improved fatigue levels, particularly physical fatigue, as assessed using the Fatigue Severity Scale. No evidence of toxicity was observed during the course of this study [30].

HemoHIM has demonstrated remarkable efficacy in mitigating immune and hematopoietic damage, specifically by restoring the Th1/Th2 balance, which is attributed to increased IL-12p70 production by antigen-presenting cells and enhanced natural killer cell activity [1]. Furthermore, HemoHIM exhibits protective effects against various conditions, including UV-induced skin damage [31], immune system impairment caused by anticancer drugs (e.g., cisplatin) [4], immune modulation associated with aging and stress [15], and inflammation [2]. Notably, a study on aged mice confirmed that HemoHIM improved immune cell function and promoted cytokine production [1]. Based on these findings, HemoHIM (Recognition Number: 2006-17) has been approved by the MFDS as a health-functional food ingredient for immunomodulation and has successfully been commercialized.

This study investigated the acute, repeat-dose oral toxicity, and genotoxicity of HemoHIM according to the OECD Test Guidelines. HemoHIM did not induce point mutations in the form of revertant colonies up to a concentration of 5,000 μg/plate, with or without metabolic activation, hence it is considered as non-mutagenic in bacterial reverse mutation test in *S. typhimurium* and *E. coli* tester strains. The results of the chromosome aberration assay indicated that HemoHIM did not induce the structural chromosome aberrations in cultured human peripheral blood lymphocytes treated up to a concentration of 625 μg/mL under short-term (+S9 and −S9) and continuous exposure (−S9) conditions. Furthermore, HemoHIM is considered non-clastogenic. Based on the in vivo micronucleus test, it was concluded that HemoHIM did not induce micronuclei formation in the polychromatic erythrocytes of the bone marrow of male and female mice treated with up to 2,000 mg/kg.

The acute oral LD50 value of HemoHIM was >2,000 mg/kg in male and female Sprague–Dawley rats. According to the UN GHS classification system, it is classified as Category 5 (possibly harmful if swallowed). For food, it is necessary to examine the acute toxicity of up to 5,000 mg/kg, rather than 2,000 mg/kg, to ensure safety. In addition, the No Observed Adverse Effect Level for the 28-day and 13-week repeat dose toxicities of HemoHIM was >2,000 mg/kg/day in Sprague–Dawley rats. Collectively, our study demonstrated that HemoHIM is safe for use as a functional food ingredient, although further studies with higher doses for acute toxicity and long-term repeated-dose toxicity are necessary.

## Data Availability

The datasets used and/or analyzed during the current study available from the corresponding author on reasonable request.
